# Delayed Bile Duct Injury Preceded by Sepsis After Biliary Cooling–Assisted Ablation of Hilar Hepatocellular Carcinoma

**DOI:** 10.14309/crj.0000000000002152

**Published:** 2026-06-03

**Authors:** Chihiro Tarumi, Yuki Tokuda, Akira Nishio, Tairyu Sato, Shogo Nagahama, Takayuki Matsumae, Koki Yamada, Yuki Nishiura, Kumi Higashihara, Tadashi Kegasawa, Aya Ishimi, Satoshi Hiyama, Katsumi Yamamoto, Chihiro Yamanaka, Toshiaki Kitayama, Hiroshi Wada, Nobuyuki Tatsumi, Akira Kaneko

**Affiliations:** 1 Department of Gastroenterology and Hepatology, Japan Community Health Care Organization, Osaka Hospital, Osaka, Japan; 2 Department of Surgery, Japan Community Health Care Organization, Osaka Hospital, Osaka, Japan; 3 Department of Radiology, Japan Community Health Care Organization, Osaka Hospital, Osaka, Japan

**Keywords:** hepatocellular carcinoma, hilar HCC, microwave thermosphere ablation, Emprint, intraductal cooling, endoscopic nasobiliary drainage, bile duct injury, bile duct stricture, sepsis, septic shock

## Abstract

An 80-year-old man with hepatitis C was diagnosed with a 12-mm hilar hepatocellular carcinoma and underwent microwave thermosphere ablation with intraductal cooling via endoscopic nasobiliary drainage. The ablation zone was adequate and no early recurrence occurred, but he developed septic shock 6 weeks later without overt biliary dilation. *Enterobacter cloacae* was isolated and treated successfully. Follow-up imaging revealed biliary dilation, consistent with delayed bile duct injury. In retrospect, this suggests that subclinical ductal injury had preceded and ultimately manifested as septic shock, likely of biliary origin. Delayed biliary injury may occur despite cooling, underscoring the need for long-term surveillance.

## INTRODUCTION

Radiofrequency ablation (RFA) is an established curative treatment for early-stage hepatocellular carcinoma (HCC).^1–3^ However, ablating tumors near the hepatic hilum remains challenging because of heat-sink effects and the risk of bile duct injury.^4–6^ To mitigate these complications, intraductal cooling via endoscopic nasobiliary drainage (ENBD) has been applied, although not completely protective.^7–9^

Microwave ablation offers advantages over RFA, including higher temperatures, faster treatment, and reduced heat-sink susceptibility. The Emprint system uses Thermosphere technology to deliver consistently large, spherical ablation zones (microwave thermosphere ablation [MTA]).^4^ These features make MTA an attractive treatment option for tumors in challenging locations such as the hepatic hilum.

We report a case of hilar HCC treated with MTA under intraductal cooling. Although initial treatment was successful, the patient later developed delayed bile duct injury. Notably, septic shock, likely of biliary origin, preceded radiologic evidence of ductal damage, underscoring that infection can herald evolving injury before imaging.

## CASE REPORT

**Figure 1.**
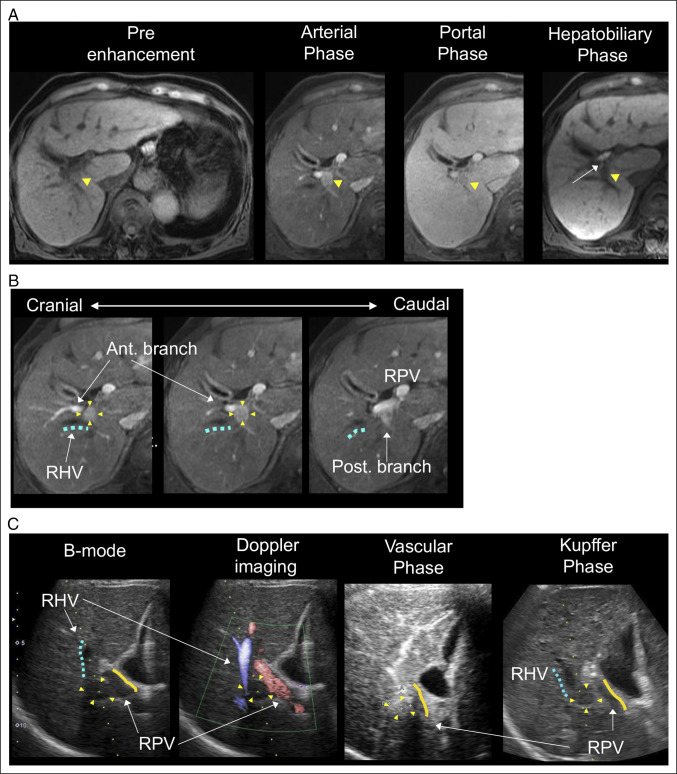
Preablation imaging of hilar HCC highlighting vascular and biliary anatomy. (A) Gadoxetic acid–enhanced magnetic resonance imaging shows a 12-mm HCC adjacent to the RPV branches and RHV. The lesion (arrowheads) demonstrates typical arterial enhancement and washout, whereas the hepatobiliary phase highlights its proximity to the right posterior segmental bile duct (arrow). (B) Sequential axial images illustrate the cranial-to-caudal relationship between the tumor (arrowheads) and adjacent vascular structures, including the RHV (blue dotted line) and anterior/posterior branches of the RPV. (C) Preablation ultrasonography demonstrates the tumor on B-mode, confirms vascular landmarks (RHV, RPV) on Doppler imaging, and shows characteristic hyperenhancement in the Sonazoid vascular phase followed by a defect in the Kupffer phase—findings that support a feasible puncture route for microwave thermosphere ablation. The tumor, RHV, and RPV are indicated by arrowheads, a blue dotted line, and an orange line, respectively. HCC, hepatocellular carcinoma; RHV, right hepatic vein; RPV, right portal vein.

An 80-year-old man with chronic hepatitis C was diagnosed with a 12-mm hilar HCC on surveillance. Baseline laboratory tests before ablation showed preserved liver function, with total bilirubin 0.8 mg/dL, aspartate aminotransferase 27 U/L, alanine aminotransferase 15 U/L, alkaline phosphatase 140 U/L, and γ-glutamyl transpeptidase 19 U/L. Gadoxetic acid–enhanced magnetic resonance imaging showed a hilar tumor abutting the right portal and hepatic veins, close to the right posterior segmental bile duct (Figure 1). Ultrasonography confirmed typical HCC enhancement and a feasible puncture route (Figure 1).

Given the hilar location, the risk of bile duct injury and incomplete ablation because of the heat-sink effect were concerns. After multidisciplinary discussion, MTA (Emprint; Medtronic) combined with intraductal cooling via ENBD was selected to balance curative intent with safety.^6,7,9^

**Figure 2.**
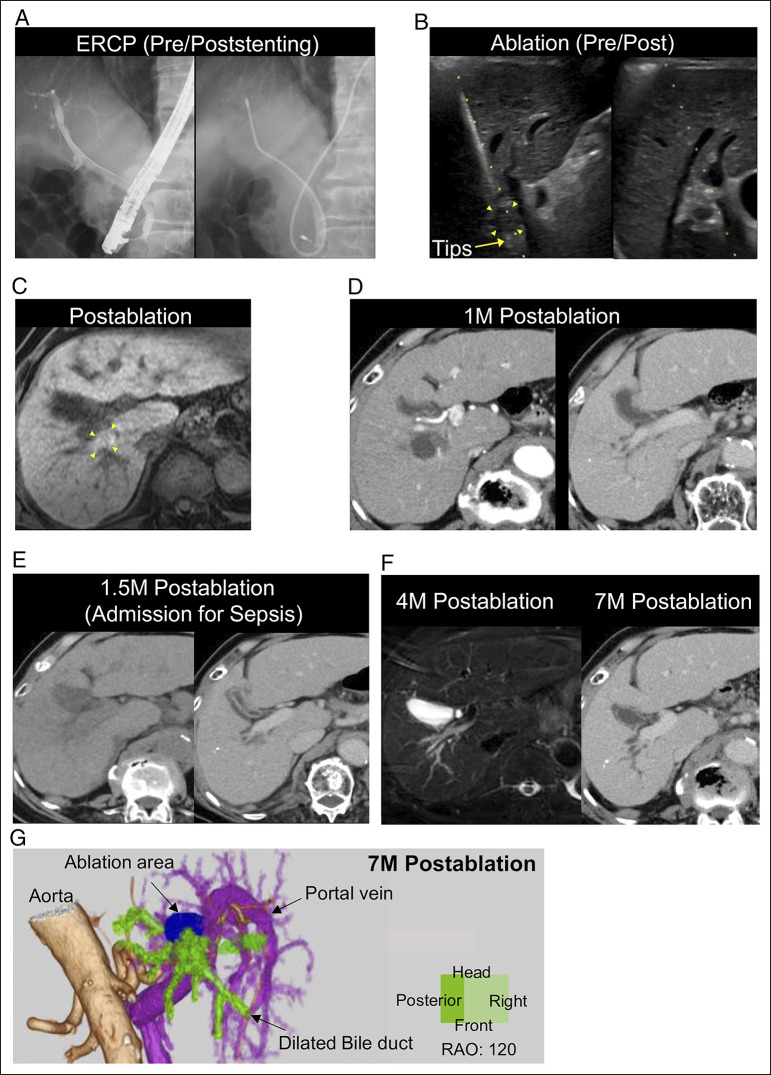
Procedure and imaging course of delayed bile duct injury after hilar HCC ablation. (A) Endoscopic retrograde cholangiopancreatography before and after placement of a 5Fr endoscopic nasobiliary drainage tube into the right anterior segmental duct for intraductal cooling. (B) Ultrasonography obtained during ablation (left) shows the Emprint antenna positioned at the tumor margin (arrowheads), whereas the postablation image (right) confirms an adequate ablation zone without immediate complications. (C) T1-weighted MRI immediately after treatment demonstrates an ablation zone with adequate margin (arrowheads). (D) Contrast-enhanced CT at 1 month shows no local recurrence and no appreciable bile duct dilation. (E) At 1.5 months (≈6 weeks) postablation: left, noncontrast CT at admission for fever; right, contrast-enhanced CT (portal phase) performed during septic shock the following evening. Both show no appreciable biliary dilation. (F) At 4 months postablation, T2-weighted MRI demonstrates definite biliary dilation, which persists at 7 months on contrast-enhanced CT (portal phase). (G) A 3D reconstruction from the 7-month postablation CT (right anterior oblique 120° view) depicts the ablation zone (blue), the biliary tree (yellow-green), portal vein branches (purple), and hepatic arteries (light brown). The biliary narrowing is located at the caudal margin of the ablation zone, anatomically correlating with the progressive ductal dilation. CT, computed tomography; ERCP, endoscopic retrograde cholangiopancreatography; MRI, magnetic resonance imaging.

On the day of ablation, endoscopic retrograde cholangiopancreatography was performed, and a 5 Fr ENBD tube was placed into the right anterior segmental duct, because of acute posterior angulation of the duct (Figure 2). Intraductal cooling with continuous saline irrigation was applied, and an Emprint antenna (20 cm) was positioned at the tumor's lower margin (Figure 2, left). Ablation at 60 W for 1 minute followed by 75 W for 3 minutes created an ablation zone with adequate margins and no immediate complications (Figure 2). The patient was discharged uneventfully.

At 1 month, computed tomography (CT) showed no local recurrence and no appreciable bile duct dilation (Figure 2). He remained asymptomatic; 2 weeks later, however, he presented with fever and chills.

On admission, he had no abdominal pain. Laboratory tests showed no leukocytosis (white blood cells 1,700/μL) and mildly elevated C-reactive protein (0.41 mg/dL), whereas biliary enzymes showed only mild elevation (alkaline phosphatase 192 U/L, γ-glutamyl transpeptidase 70 U/L) (Table 1). Chest and abdominal CT showed no evidence of pneumonia or other intra-abdominal infection. Urinalysis was unremarkable, and no clinically significant skin or soft tissue infection was identified. Blood cultures grew *Enterobacter cloacae*, and empiric meropenem was started (Table 2). On day 2, he developed hypotension, rising C-reactive protein (16.9 mg/dL), and acute kidney injury (creatinine 1.3 mg/dL), consistent with septic shock, requiring short-term catecholamine support. He stabilized with antibiotics and was discharged on day 16. Given the recent hilar ablation, a biliary source—including cholangitis—was considered; however, imaging at admission and during deterioration showed no overt biliary dilation and subtle prominence was not appreciated prospectively (Figure 2); thus, the etiology remained unclear.

**Table 1.** Laboratory findings on admission at 1.5 months postablation

Complete blood count		
WBC	1.7	×10^3^/μL
NEUT	88.7	%
Ly	10.2	%
RBC	3.92	×10^6^/μL
Hb	13.3	g/dL
Platelet	11.8	×10^4^/μL

Blood coagulation test		
PT	109.5	%
PT-INR	0.96	
D-dimer	6.1	µg/mL
FDP	18.9	µg/mL

Blood chemistry		
T-bil	1.3	mg/dL
AST	44	U/L
ALT	26	U/L
ALP	192	U/L
γ-GTP	70	U/L
LDH	265	U/L
TP	7.0	g/dL
Alb	3.9	g/dL
BUN	23	mg/dL
Cr	0.9	mg/dL
CRP	0.41	mg/dL

Alb, albumin; ALP, alkaline phosphatase; ALT, alanine aminotransferase; AST, aspartate aminotransferase; BUN, blood urea nitrogen; Cr, creatinine; CRP, C-reactive protein; D-dimer, D-dimer; FDP, fibrin/fibrinogen degradation products; Hb, hemoglobin; LDH, lactate dehydrogenase; Ly, lymphocyte; NEUT, neutrophil; Platelet, platelet count; PT, prothrombin time; PT-INR, prothrombin time-international normalized ratio; RBC, red blood cell count; T-bil, total bilirubin; TP, total protein; WBC, white blood cell count; γ-GTP, γ-glutamyl transpeptidase.

**Table 2.** Antimicrobial susceptibility of *Enterobacter cloacae*

Drug class	Drug	MIC	Sensitivity
Penicillin	Ampicillin	≤8	R
β-lactam/β-lactamase inhibitor	Ampicillin/sulbactam	≤8	R
β-lactam/β-lactamase inhibitor	Piperacillin/tazobactam	≤8	S*
1st-gen cephalosporin	Cefazolin	8	R
3rd-gen cephalosporin	Ceftriaxone	≤1	S*
3rd-gen cephalosporin	Ceftazidime	2	S*
4th-gen cephalosporin	Cefepime	≤2	S
Cephamycin (oxacephem)	Cefmetazole	≤16	R
Carbapenem	Meropenem	≤0.12	S
Fluoroquinolone	Levofloxacin	≤0.12	S
Aminoglycoside	Gentamicin	≤4	S
Aminoglycoside	Amikacin	≤16	S

MIC, minimum inhibitory concentration; R, resistant; S, susceptible; S*, susceptible in vitro but risk of resistance during therapy because of AmpC β-lactamase induction.

At approximately 2 and 5 months after discharge (corresponding to 4 and 7 months postablation), imaging showed no local recurrence but progressive biliary dilation, raising concern for evolving ductal injury (Figure 2). 3D reconstruction at 7 months postablation demonstrated a focal stricture along the peripheral (caudal) margin of the ablation zone, resulting in upstream biliary dilation (Figure 2). In retrospect, these findings suggest that an unrecognized, evolving bile duct injury may have already been present during the septic episode, potentially contributing to biliary infection despite the absence of overt radiologic abnormalities at that time.

## DISCUSSION

This report highlights diagnostic and management challenges after hilar HCC ablation with intraductal cooling. Septic shock occurred at a time when CT showed no definite biliary dilation and laboratory findings did not suggest cholangitis; on retrospective review, however, subtle ductal prominence could not be excluded. This sequence suggests that infection may precede overt imaging changes of evolving bile duct injury.

Possible mechanisms include thermal spread beyond the effectively cooled bile duct lumen, ischemic injury to the peribiliary vascular plexus, and delayed fibrotic remodeling, which together may result in progressive bile duct stricture formation weeks to months after ablation.^10^

Treatment of hilar HCC must balance oncologic control with protection of central bile ducts.^6,8^ Because MTA achieves rapid high temperatures and is less susceptible to heat-sink than RFA, central bile ducts remain at risk; therefore, intraductal cooling is often used for treating hilar lesions.^4,11–13^ Previous studies on hilar HCC treated with RFA consistently show cooling reduces biliary complications without increasing recurrence, although not completely protective, as illustrated in our case.^7–9,14^ To our knowledge, reports of MTA combined with intraductal cooling for hilar HCC remain scarce. Importantly, bile duct injury can compromise future locoregional therapy—biloma formation increases infection risk and may preclude repeat ablation or transcatheter arterial chemoembolization near the injured duct; persistent stricture can lead to segmental atrophy, impaired liver function, and a narrowed therapeutic window.^10,15^

As an alternative, stereotactic body radiotherapy (SBRT) has been proposed for centrally located HCC where thermal ablation carries high risk.^16,17^ However, biliary toxicity remains concerning; late grade ≥3 complications occurred at similar rates after SBRT and RFA (3.3% vs 6%, *P* = 0.38).^18^ In addition, post-SBRT imaging interpretation can be difficult because robust imaging criteria for local recurrence remain limited, which may delay recognition and retreatment.^19^

Given the lack of established guidelines, this case underscores the importance of close postprocedural surveillance after hilar ablation. Serial monitoring of biliary enzymes and interval imaging may facilitate earlier recognition of delayed bile duct injury. Importantly, unexplained sepsis after hilar ablation should raise suspicion for occult biliary injury.

## DISCLOSURES

Author contributions: C. Tarumi, Y. Tokuda, and A. Nishio contributed equally to drafting the manuscript and curating clinical data. C. Tarumi, Y. Tokuda, A. Nishio, T. Sato, S. Nagahama, T. Matsumae, K. Yamada, Y. Nishiura, K. Higashihara, T. Kegasawa, A. Ishimi, S. Hiyama, K. Yamamoto, C. Yamanaka, T. Kitayama, H. Wada, N. Tatsumi, and A. Kaneko contributed to patient care, clinical data interpretation, and critical revision of the manuscript. A. Kaneko provided senior supervision and conducted the final critical review of the manuscript. All authors approved the final version of the manuscript and agree to be accountable for all aspects of the work. Y. Tokuda is the article guarantor.

Financial disclosure: None to report.

Informed consent was obtained for this case report.

## ABBREVIATIONS:

CT: computed tomography
ENBD: endoscopic nasobiliary drainage
HCC: hepatocellular carcinoma
MRI: magnetic resonance imaging
MTA: microwave thermosphere ablation
RFA: radiofrequency ablation
SBRT: stereotactic body radiotherapy
